# Reclassification of Heart Failure with Preserved Ejection Fraction Following Cardiac Sympathetic Nervous System Activation: A New Cutoff Value of 58%

**DOI:** 10.3390/tomography8030132

**Published:** 2022-06-18

**Authors:** Toshihiko Goto, Takafumi Nakayama, Junki Yamamoto, Kento Mori, Yasuhiro Shintani, Shohei Kikuchi, Hiroshi Fujita, Hidekatsu Fukuta, Yoshihiro Seo

**Affiliations:** 1 Department of Cardiology, Nagoya City University Graduate School of Medical Sciences, Nagoya 467-8601, Japan; jun_yama@med.nagoya-cu.ac.jp (J.Y.); kento-m@med.nagoya-cu.ac.jp (K.M.); shintany@med.nagoya-cu.ac.jp (Y.S.); syohey@med.nagoya-cu.ac.jp (S.K.); fujipon@med.nagoya-cu.ac.jp (H.F.); yo-seo@med.nagoya-cu.ac.jp (Y.S.); 2 Department of Cardiovascular Medicine, Nagoya City University West Medical Center, Nagoya 462-8508, Japan; tnakayama83@gmail.com; 3 Clinical Research Management Center, Nagoya City University Hospital, Nagoya 467-8601, Japan; fukuta-h@med.nagoya-cu.ac.jp

**Keywords:** cardiac sympathetic nervous system, heart failure, left ventricular ejection fraction, metaiodobenzylguanidine, mortality

## Abstract

Heart failure (HF) with preserved left ventricular ejection fraction (LVEF) is a heterogeneous syndrome. An LVEF of 50% is widely used to categorize patients with HF; however, this is controversial. Previously, we have reported that patients with an LVEF of ≥ 58% have good prognoses. Further, cardiac sympathetic nervous system (SNS) activation is a feature of HF. In this retrospective, observational study, the cardiac SNS activity of HF patients (*n* = 63, age: 78.4 ± 9.6 years; male 49.2%) with LVEF ≥ 58% (*n* = 15) and LVEF < 58% (*n* = 48) were compared using ^123^I-metaiodobenzylguanidine scintigraphy. During the follow-up period (median, 3.0 years), 18 all-cause deaths occurred. The delayed heart/mediastinum (H/M) ratio was significantly higher in the LVEF ≥ 58% group than in the LVEF < 58% group (2.1 ± 0.3 vs. 1.7 ± 0.4, *p* = 0.004), and all-cause mortality was significantly lower in patients in the former than those in the latter group (log-rank, *p* = 0.04). However, when these patients were divided into LVEF ≥ 50% (*n* = 22) and LVEF < 50% (*n* = 41) groups, no significant differences were found in the delayed H/M ratio, and the all-cause mortality did not differ between the groups (log-rank, *p* = 0.09). In conclusion, an LVEF of 58% is suitable for reclassifying patients with HF according to cardiac SNS activity.

## 1. Introduction

In clinical settings, patients with heart failure (HF) are generally divided into two main categories based on the 50% cutoff value for left ventricular ejection fraction (LVEF): HF with preserved LVEF (HFpEF, LVEF ≥ 50%) and HF with reduced LVEF (HFrEF, LVEF < 50%). Recently, it has become increasingly common to refer to HF with an LVEF of 40–50% as HF with mid-range EF (HFmrEF) [1]. However, using an LVEF of 50% as the cutoff is controversial; no effective treatments have been demonstrated to lead to improved survival in patients with HFpEF when the conventional cutoff value of 50% is used in HFpEF. This is because HFpEF is a heterogeneous syndrome, and an LVEF of 50% may not be an ideal cutoff value for the treatment of HF. In contrast, an activated cardiac sympathetic nervous system (SNS) is one of the features of HF [2]. ^123^I-metaiodobenzylguanidine (MIBG) is the most widely used imaging agent for evaluating cardiac SNS abnormalities [3,4,5]. MIBG is an analog of norepinephrine and shares the same uptake, storage, and release systems at nerve endings with norepinephrine [6]. An increase in norepinephrine turnover and pre-synaptic norepinephrine deficits result in increased washout rate (WR) from the heart and decreased MIBG activity, which is quantified as the heart to mediastinum (H/M) ratio. Therefore, it has been established that elevated cardiac SNS activation, evaluated using ^123^I-MIBG, is an indicator of prognosis in patients with chronic HF [7,8,9]. Furthermore, a recent study has also reported the prognostic value of cardiac ^123^I-MIBG imaging in patients with acute decompensated HF [10]. Previously, we reported that patients with an LVEF of ≥58% have good prognoses [11]. The inertia stress of late systolic aortic flow, which is defined from the left ventricular (LV) pressure (P)—the first derivative of LV pressure (dP/dt) relation, as we reported previously [12]—is produced by left ventricles with good systolic function [13]. Therefore, the lack of inertia stress is related to the loss of elastic recoil in the left ventricle, which in turn results in the deterioration of LV relaxation [12]. This means that even with an LVEF of ≥50%, the left ventricle would not have good LV systolic function if it does not have inertia stress. Therefore, a left ventricle with inertia stress means that the left ventricle has both good systolic and diastolic functions. In our previous study, an LVEF of ≥58% was found to be a surrogate indicator that the left ventricle has the inertia stress of late systolic aortic flow [11]. Thus, the primary aim of this study was to assess the validity of a new HF cutoff value of LVEF at 58% in terms of cardiac SNS activation. The secondary aim was to investigate the association between an LVEF of 58% and prognosis.

## 2. Materials and Methods

### 2.1. Study Population

This was a single-center, retrospective, observational study. We collected data on HF patients admitted to Nagoya City University Hospital because of acute decompensated HF between October 2013 and August 2018. A total of 63 consecutive patients with HF who underwent simultaneous MIBG scintigraphy and comprehensive echocardiography during the stable phase after HF treatment and before discharge were eligible for this study. Patients with autonomic nervous system abnormality, such as Parkinson’s disease, were excluded. HF was diagnosed based on the modified Framingham criteria [14]. The etiology of HF and the cause of decompensation were defined as ischemic, non-ischemic, hypertensive, valvular, or abnormal heart rhythm etiology based on the diagnosis by the attending physician. Plasma B-type natriuretic peptide (BNP) level and New York Heart Association (NYHA) classification were evaluated at the same time. All-cause mortality and a composite of all-cause mortality and HF readmission were investigated in this study.

### 2.2. Cardiac ^123^I-MIBG Scintigraphy

Patients that were stable after HF treatment underwent MIBG imaging for assessing cardiac SNS activity using standard procedures. Anterior planar images using scinticameras equipped with low-energy-type collimators were obtained 15–30 min (early phase) and 3–4 h (delayed phase) after administering 111 MBq ^123^I-MIBG (Fujifilm RI Pharma Co., Ltd., Tokyo, Japan). Early and delayed H/M ratios were calculated from the mean count of the whole heart and the upper third of the mediastinum in the planar anterior view. The WR was also calculated using the following equation:

*WR* = (*early*
*heart*
*counts* − *delayed*
*heart*
*counts*)/*early*
*heart*
*counts*.

### 2.3. Statistical Analysis

SPSS statistical software (version 23.0, SPSS Inc., Chicago, IL, USA) was used for all statistical analyses. Continuous variables were presented as the mean ± standard deviation (SD) for normally distributed variables and the median and interquartile range (IQR) for non-normally distributed variables. Categorical variables were summarized as frequencies (%). Regarding the comparison of the two groups, continuous variables were compared using an unpaired Student’s *t*-test for normally distributed variables and the Mann–Whitney U test for non-normally distributed variables. Differences in prevalence between the two groups were compared using the chi-squared test. The ability of the delayed H/M ratio to identify an LVEF of 58% or 50% was evaluated using a receiver operating characteristic (ROC) curve analysis, in which the area under the curve (AUC) and 95% confidence intervals (CIs) were calculated. Variables in more than two groups were evaluated using an analysis of variance (ANOVA) with Bonferroni adjustment for normally distributed variables.

For endpoint-free survival analysis, Kaplan–Meier curves were generated and compared using the log-rank test. Differences with *p* < 0.05 were considered statistically significant.

## 3. Results

### 3.1. Clinical Characteristics of Study Patients

A total of 63 patients (age, 78.4 ± 9.6 years; male patients, 49.2%) were investigated. The clinical characteristics of all patients and the demographics of the subgroups when divided according to LVEF (58% or 50%) are presented in Table 1.

### 3.2. HF Cutoff Value of LVEF 58%

The LVEF was significantly different between the LVEF ≥ 58% (*n* = 15) and LVEF < 58% groups (*n* = 48) (70.4 ± 8.3% vs. 36.6 ± 11.1%, *p* < 0.001). Heart rate tended to be lower in patients with an LVEF of ≥58% than in those with an LVEF of <58%; however, this difference was not significant. The etiology of HF is presented in Table 2. The prevalence of HF due to an ischemic (0.0% vs. 25.0%, *p* = 0.03) or non-ischemic cardiomyopathy etiology (26.7% vs. 56.3%, *p* = 0.045) was lower in patients with an LVEF of ≥58% than in those with an LVEF of <58%, whereas the prevalence of abnormal heart rhythm etiology, including both tachyarrhythmia and bradyarrhythmia, was significantly higher in patients with an LVEF of ≥58% than in those with an LVEF of <58% (46.7% vs. 10.7%, *p* = 0.002). Patients with an LVEF of ≥58% had a higher prevalence of permanent atrial fibrillation than those with an LVEF of <58% (86.7% vs. 52.1%, *p* = 0.02).

### 3.3. HF Cutoff Value of LVEF 50%

When patients were divided according to an LVEF of 50% (LVEF ≥ 50%, *n* = 22; LVEF < 50%, *n* = 41), the clinical characteristic trends were similar to those noted when the patients were divided according to an LVEF of 58%. LVEF was significantly different between the LVEF ≥ 50% and the LVEF < 50% groups (65.4 ± 10.2% vs. 33.5 ± 8.9%, respectively, *p* < 0.001). Regarding the etiologies of HF, no differences in ischemic etiologies were confirmed between the two groups. Hypertensive etiologies were significantly more common in patients with an LVEF of ≥50% than in those with an LVEF of <50% (13.6% vs. 0.0%, respectively, *p* = 0.02).

### 3.4. Medication

Cardiac medications administered to the patients are also presented in Table 3. The use of β-blockers did not differ between patients with an LVEF of ≥58% and those with an LVEF of <58% (53.3% vs. 70.8%, respectively *p* = 0.21). In contrast, the use of β-blockers was significantly lower in patients with an LVEF of ≥50% than in those with an LVEF of <50% (50.0% vs. 75.6%, respectively, *p* = 0.04). The details of the use of β-blockers are presented in Table 3.

### 3.5. Cardiac SNS Activity

**Figure 1:**
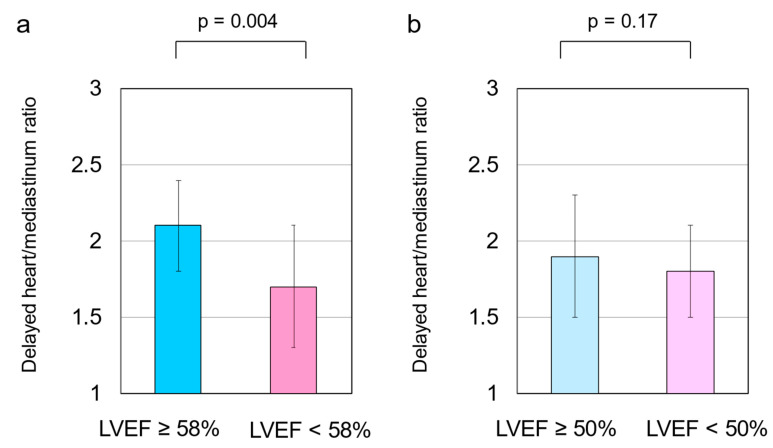
(**a**) The delayed heart/mediastinum (H/M) ratio was significantly higher in patients with a left ventricular ejection fraction (LVEF) of ≥58% than in those with an LVEF of <58% (2.1 ± 0.3 vs. 1.7 ± 0.4, *p* = 0.004). (**b**) When divided into two groups using an LVEF of 50%, no significant differences were found in the delayed H/M ratio between the two groups (1.9 ± 0.4 vs. 1.8 ± 0.3, *p* = 0.17). These data were compared using an unpaired Student’s *t*-test.

**Figure 2:**
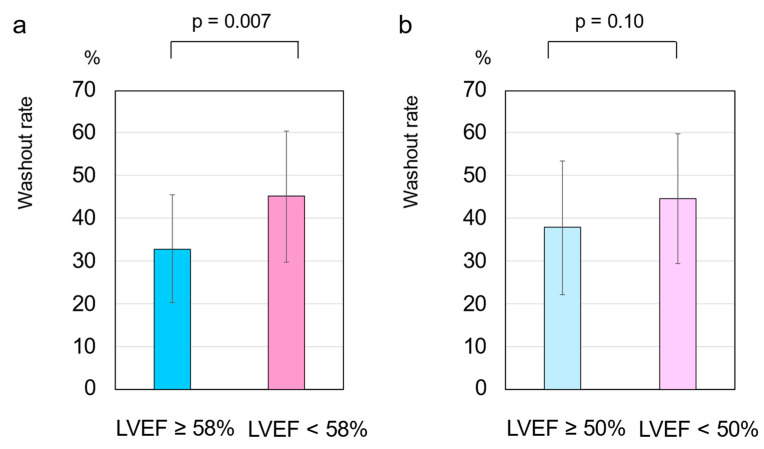
(**a**) The washout rate (WR) was significantly lower in patients with a left ventricular ejection fraction (LVEF) of <58% compared to that of those with an LVEF of ≥58% (32.9 ± 12.6% vs. 45.1 ± 15.4%, *p* = 0.007). (**b**) No significant differences were found in the WR between patients with an LVEF of ≥50% and those with an LVEF of <50% (37.8 ± 15.7% vs. 44.6 ± 15.2%, *p* = 0.10). These data were compared using an unpaired Student’s *t*-test.

**Figure 3:**
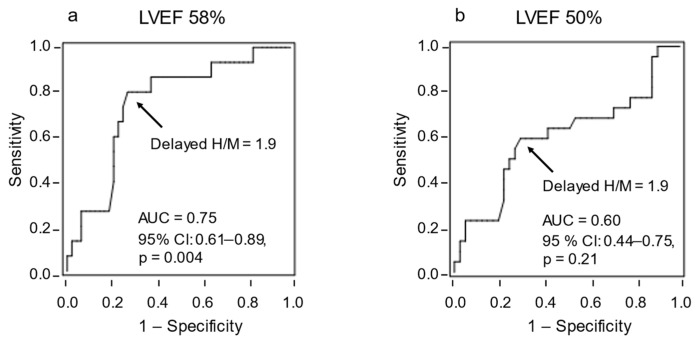
The ability of the delayed heart/mediastinum (H/M) ratio to identify LVEF of 58% or 50% was evaluated using a receiver operating characteristic (ROC) curve analysis, in which the area under the curve (AUC) and 95% confidence interval (CI) were calculated. (**a**) The ROC curve for the delayed H/M ratio for predicting a left ventricular ejection fraction (LVEF) of 58% was 0.75 (95% CI 0.61–0.89, *p* = 0.004). From this analysis, a delayed H/M ratio of 1.9 had a sensitivity and specificity of 80.0% and 72.9%, respectively, for predicting an LVEF of 58%. (**b**) The area under the ROC curve for delayed H/M ratio for predicting an LVEF of 50% was not significant (0.60; 95% CI 0.44–0.75, *p* = 0.21).

**Figure 4:**
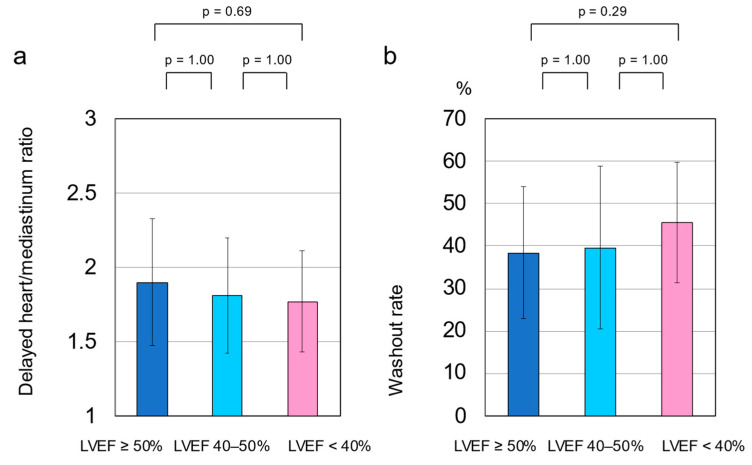
Comparison of the delayed heart/mediastinum ratio (H/M) and the washout rate (WR) among the three groups; LVEF ≥ 50% (*n* = 22), LVEF 40–50% (*n* = 9), and LVEF < 40% (*n* = 32). No significant differences were observed in the delayed H/M ratio (1.9 ± 0.4 vs. 1.8 ± 0.4 vs. 1.8 ± 0.3, respectively, *p* = 0.48 (**a**) or WR (37.8 ± 15.7% vs. 39.7 ± 19.2%, vs. 45.5 ± 14.3%, respectively, *p* = 0.22 (**b**) among the three groups. These data were compared using an analysis of variance (ANOVA) with Bonferroni adjustment.

The delayed H/M ratio was 1.82 ± 0.4, and the WR was 42.2 ± 15.6% in the total cohort. The delayed H/M ratio was significantly higher in patients with an LVEF of ≥58% than in those with an LVEF of <58% (2.1 ± 0.3 vs. 1.7 ± 0.4, respectively, *p* = 0.004) (Figure 1a). The WR was significantly lower in patients with an LVEF of ≥58% than in those with an LVEF of <58% (32.9 ± 12.6% vs. 45.1 ± 15.4%, respectively, *p* = 0.007) (Figure 2a). However, no significant differences were observed in the delayed H/M ratio or WR between patients with an LVEF of ≥50% and those with an LVEF of <50% (Figure 1b and Figure 2b). The area under the ROC curve for delayed H/M ratio for predicting an LVEF of 58% was 0.75 (95% CI, 0.61–0.89; *p* = 0.004). From this analysis, a delayed H/M ratio of 1.9 had a sensitivity and specificity of 80.0% and 72.9%, respectively, for predicting an LVEF of 58% (Figure 3a). In contrast, the area under the ROC curve for delayed H/M ratio for predicting an LVEF of 50% was not significant (AUC = 0.60; 95% CI 0.44–0.75, *p* = 0.21) (Figure 3b). Then, patients were divided into three groups; LVEF ≥ 50% (*n* = 22), LVEF 40–50% (*n* = 9), and LVEF < 40% (*n* = 32). No significant differences were observed in the delayed H/M ratio (1.9 ± 0.4 vs. 1.8 ± 0.4 vs. 1.8 ± 0.3, respectively, *p* = 0.48; Figure 4a) or the WR (37.8 ± 15.7% vs. 39.7 ± 19.2%, vs. 45.5 ± 14.3%, respectively, *p* = 0.22; Figure 4b) among the three groups.

### 3.6. Differences in LVEF and Cardiac SNS Activity by Gender

As shown in Table 1, there were no significant differences in sex when all patients were divided by an LVEF of 58% or an LVEF of 50%. Furthermore, no significant differences were found in the LVEF (42.8 ± 18.4% vs. 46.4 ± 17.5%, *p* = 0.46), the delayed H/M ratio (1.9 ± 0.4% vs. 1.8 ± 0.4, *p* = 0.51), or the WR (38.8 ± 14.2% vs. 45.5 ± 16.3%, *p* = 0.09) between males (*n* = 31) and females (*n* = 32). These data were compared using an unpaired Student’s *t*-test. Table 4 shows the LVEF, the delayed H/M ratio, and the WR between males and females using the cutoff value of LVEF at 58% or LVEF at 50%. There were no significant differences in the LVEF, the delayed H/M ratio, or the WR between males and females at either cutoff value of LVEF.

### 3.7. All-Cause Mortality and the Combined Endpoint

**Figure 5:**
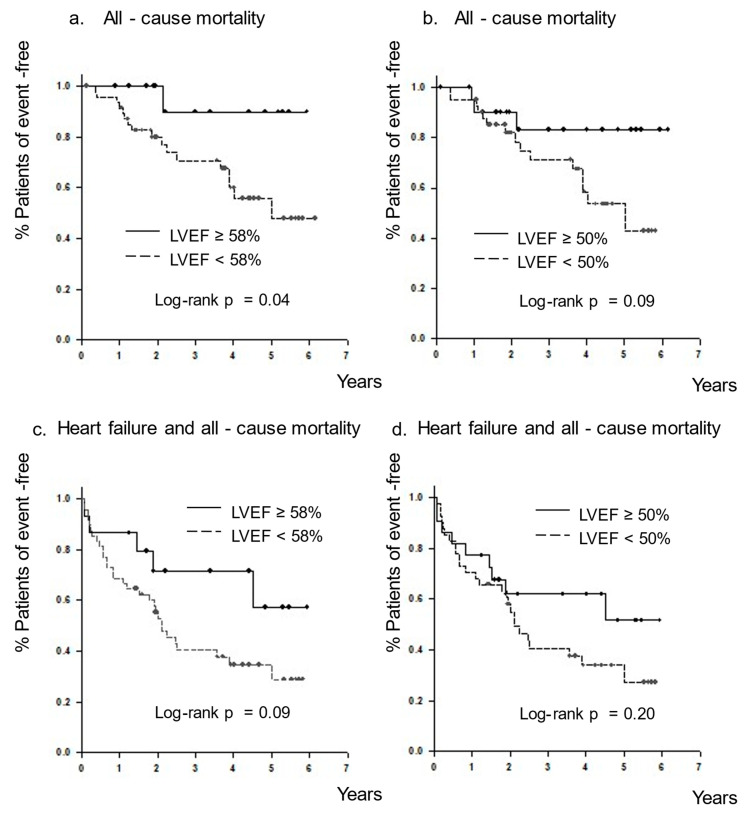
For endpoint-free survival analysis, Kaplan–Meier curves were generated and compared using the log-rank test. Kaplan–Meier curves for all-cause mortality in patients with a left ventricular ejection fraction (LVEF) of ≥58% (**a**) and ≥50% (**b**). The survival rate was significantly higher in patients with an LVEF of ≥58% than in those with an LVEF of < 58%. No significant differences were found between patients with an LVEF of ≥50% and those with <50%. Kaplan–Meier curves for the combined endpoint of subsequent heart failure and all-cause mortality in patients with an LVEF of ≥58% (**c**) and ≥50% (**d**). No significant differences were found between patients with an LVEF of ≥58% and <58%, as well as between patients with an LVEF of ≥50% and <50%.

During the follow-up period (median, 3.0 years; mean, 3.3 ± 1.8 years), 28 unscheduled hospitalizations owing to HF and 18 all-cause deaths were observed (Table 5). The causes of death were HF (*n* = 8), malignant neoplasm (*n* = 3), senility (*n* = 2), trauma (*n* = 2), sudden death (*n* = 2), and infection (*n* = 1). In the LVEF of ≥58% group, one death out of 15 patients (6.7%) was observed, compared to 15 out of 41 patients (36.6%) in the LVEF of <58% group. Out of the 10 cases of cardiac death, nine cases were in the LVEF < 50% group. The only deceased patient with an LVEF of ≥58% died of senility. The combined endpoints of HF readmission and all-cause mortality occurred in 35 out of 63 patients (55.6%). In the LVEF of ≥58% group, five out of 15 patients (33.3%) were hospitalized for HF, and in the LVEF of <50%, nineteen of 41 patients (46.3%) were hospitalized for HF. Among the seven patients with an LVEF of 50% to 58%, two deaths (one from senility and one from HF) and four HF hospitalizations were observed. The Kaplan–Meier plot showed that the incidence of all-cause mortality was significantly lower in patients with an LVEF of ≥58% than in those with an LVEF of <58% (log-rank, *p* = 0.04; Figure 5a). In contrast, all-cause mortality did not differ between patients with an LVEF of ≥50% and those with an LVEF of <50% (log-rank, *p* = 0.09; Figure 5b). Figure 5 shows the Kaplan–Meier curve for the composite of all-cause mortality and HF readmission with a cutoff of 58% LVEF (log-rank, *p* = 0.09; Figure 5c) and 50% LVEF (log-rank, *p* = 0.20; Figure 5d). Regarding the composite of all-cause mortality and HF readmission, no significant difference in event-free rate was observed when patients were divided into the two groups, both with an LVEF cutoff of 58% and 50%.

## 4. Discussion

An LVEF of 50% is widely used to categorize patients with HF; however, this is controversial. This study demonstrated that cardiac SNS activity was more elevated in patients with an LVEF of <58% than in those with an LVEF of ≥58%. Furthermore, an LVEF of <58% was significantly associated with all-cause mortality. In contrast, no significant differences were found in cardiac SNS activity in patients with an LVEF of ≥50% and those with an LVEF of <50%, and no significant relationships were observed in all-cause mortality. Furthermore, no significant differences in cardiac SNS activity were also found when patients were divided into the three groups: LVEF ≥ 50%, LVEF 40–50%, and LVEF < 40%.

No effective treatments have been shown to improve the survival of patients with HFpEF when the cutoff value of LVEF is 50%. This is because HFpEF is a heterogeneous syndrome, and its therapeutic target is elusive. Therefore, resolving the heterogeneity of HFpEF with an improved classification may lead to improved outcomes [15]. Previously, we proposed an LVEF of 58%, rather than 50%, as a cutoff value in patients with HF [11]. This is because an LVEF of ≥58% is a surrogate indicator that the left ventricle has both good systolic and diastolic functions. On the other hand, cardiac SNS is activated in order to maintain systemic hemodynamics and peripheral circulation, which is one of the features of HF. Myocardial abnormality caused by LV systolic and/or diastolic dysfunction is the main cause of HF, which can be visualized using ^123^I-MIBG imaging as activated cardiac SNS. However, the causes of HF are quite diverse. In particular, the causes of HF in patients with high LVEF are often attributed to high blood pressure, atrial fibrillation, and aortic stiffness, including ventricular–arterial coupling [1,16]. Our study demonstrated that cardiac SNS activity evaluated by ^123^I-MIBG scintigraphy was significantly lower in patients with an LVEF of ≥58% than in those with an LVEF of <58%. Therefore, an LVEF of 58% is a good cutoff value to differentiate patients with HF owing to cardiac causes from those with HF due to non-cardiac causes. In contrast, cardiac SNS activation did not differ between patients with an LVEF of ≥50% and those with an LVEF of <50%. This means that there was a mixed population of HF with and without cardiac dysfunction in patients with an LVEF of ≥50%. Furthermore, no significant differences in cardiac SNS activity were also found among the three groups, LVEF ≥ 50%, LVEF 40–50%, and LVEF < 40%. Seo et al. recently reported on the prognostic value of MIBG in acute decompensated HF, in which a low delayed H/M ratio was more frequent in patients with HFrEF and HFmrEF than in those with HFpEF, using an LVEF of 50% as the cutoff [10]. This inconsistency may have resulted from differences in the patient background between the studies, such as age and NYHA class. Our study patients were older and consisted of patients with more severe HF based on the NYHA Classes compared to those of the abovementioned study. However, it was consistent with their study that no differences in the delayed H/M ratio were found between the group of patients with an LVEF of 40–50% and those with an LVEF of <40%. Thus, our study found that there were significant differences in cardiac SNS activity when patients with heart failure were divided by an LVEF of 58%, but no significant differences when divided into two groups with an LVEF of 50%, or three groups of LVEF ≥ 50%, LVEF 40–50%, and LVEF < 40%.

No differences were found in the use of β-blockers between patients with an LVEF of ≥58% and those with an LVEF of <58%. Nevertheless, cardiac SNS was activated in patients with an LVEF of <58%, suggesting that β-blockers may be a possible treatment option in such patients. In contrast, the use of β-blockers was higher in patients with an LVEF of <50% than in those with an LVEF of ≥50%. However, no difference in cardiac SNS activation was found between the two groups, suggesting that β-blockers may also be a possible treatment option in patients with an LVEF of ≥50%. Therefore, both of these results suggest that β-blockers may have been underused in patients with an LVEF of 50–58%. The reason for the high use of β-blockers in patients with an LVEF of ≥58% may be due to the high prevalence of atrial fibrillation in this group. In our study, there were differences in the use of angiotensin-converting enzyme inhibitors when the LVEF cutoff value was either 58% or 50%. The differences could be attributed to the potential prevention of cardiac remodeling by angiotensin-converting enzyme inhibitors in the presence of systolic dysfunction. The high use of calcium channel blockers in patients with an LVEF of ≥50% is due to the higher prevalence of hypertension in this group.

There was a significant difference in all-cause mortality when the LVEF cutoff value was 58%; however, there was no significant difference in all-cause mortality when the LVEF cutoff value was 50%. We previously reported that significant differences were found in all-cause mortality and subsequent HF when the LVEF cutoff value was 58% [11]. However, these differences in the composite all-cause mortality and HF readmission were not found in this study. This discrepancy may have resulted from differences in the characteristics of the target patients, such as age and underlying disease. This may also be partly because of the higher incidence of subsequent HF hospitalizations compared to the previous study. In this present study, the proportion of deaths and HF hospitalizations in the LVEF of 50–58% group was higher than in the LVEF > 58% group, as in the LVEF < 50% group. Therefore, a larger number of patients may have led to a different conclusion on the composite endpoint.

In previous studies using ^123^I-MIBG, it has already been established that activated cardiac SNS is an indicator of prognosis in both chronic HF and acute decompensated HF [7,8,9,10], and the findings of our study are consistent with those of previous studies. The ADMIRE-HF study, which prospectively assessed the event rates in patients with symptomatic HF using ^123^I-MIBG, showed significantly lower event rates in the delayed H/M ratio ≥ 1.60 group than in the delayed H/M ratio < 1.60 group [7]. A Japanese pooled study also reported that a delayed H/M ratio of <1.68 was a prognostic indicator of lower survival (Nakata et al. 2013). Furthermore, a delayed H/M ratio of >2.0 has been reported to have a low risk of cardiac mortality (<5%/5 years) [8]. In our study, the delayed H/M ratio for detecting an LVEF of 58% was 1.9, and no patients with an LVEF of >58% died from cardiac causes.

We believe that an LVEF cutoff of 58% is a good candidate to reclassify patients with HFpEF based on cardiac SNS activation. The use of β-blockers for the treatment of HF patients with an LVEF of 50–58% should also be reconsidered. While there have been meta-analyses showing the potential efficacy of β-blockers in HFpEF cases [17,18], positive outcomes of β-blockers have not been reported in HFpEF treatment [19,20]. One possible reason for the ineffectiveness of β-blockers in patients with HFpEF is the existence of chronotropic incompetence. Chronotropic incompetence is the inability of the heart to increase its rate of contraction with increased activity and is an independent predictor of overall mortality [21,22]; furthermore, β-blockers, in the presence of chronotropic incompetence, prevent a compensatory increase in heart rate. Atrial fibrillation is common in patients with HFpEF [23], and we previously reported the relationship between an increase in heart rate and exercise tolerance in patients with atrial fibrillation with preserved LVEF; an adequate increase in heart rate is important to maintain exercise tolerance in such patients [24]. It has also been reported that lenient heart rate control is as effective as strict heart rate control in patients with permanent atrial fibrillation [25]. Therefore, it is understandable that a compensatory increase in heart rate would be needed, especially in patients with HF. In our study, heart rate tended to be lower in patients with an LVEF of ≥58% than in those with an LVEF of <58%, despite the higher prevalence of atrial fibrillation. These findings indicate that β-blockers may have been overused in the treatment of patients with an LVEF of ≥58%. Therefore, a reduction in the use of β-blockers should be considered, especially in patients with atrial fibrillation and an LVEF of > 58%. In contrast, β-blockers may be useful in patients with atrial fibrillation and an LVEF of 50–58% because the activated cardiac SNS could be a therapeutic target. We have recently reported that β-blockers may be beneficial in HFpEF patients with atrial fibrillation [26]. Thus, future studies are needed for validating the reclassification of HFpEF with an LVEF of 58%, and to examine the usefulness of β-blockers in patients with an LVEF of 50–58%, especially those with atrial fibrillation. If the usefulness of β-blockers is demonstrated, it would have a significant impact on the management and treatment of such patients in daily clinical practice.

This study has a few limitations. First, this was a single-center, retrospective, observational study that included a limited number of patients. Particularly when divided into the three groups, the number of patients with an LVEF of 40–50% was only nine. The impact of gender differences on the results of this study could also not be examined. Although the cutoff values of LVEF currently used in daily clinical practice are not set separately by gender, further study is needed to investigate the impact of gender differences on the LVEF or the cardiac SNS activity to understand the pathophysiology of heart failure patients. Second, the same cutoff value of LVEF at 58% was used, even though the targeted patients were different from those in our previous study. However, the left ventricle, which preserves the inertia stress of late systolic aortic flow with good left ventricle systolic and diastolic function, is not likely to be different depending on the underlying heart disease. Therefore, we believe that an LVEF of 58% is a reliable value. Third, the age of the study patients was older compared to other similar previous studies [10]. Therefore, it should be noted that the results of this study are not applicable to all elderly patients.

## 5. Conclusions

Cardiac SNS activity was more elevated in patients with an LVEF of <58% than in those with an LVEF of ≥58%. Furthermore, an LVEF of <58% was significantly associated with all-cause mortality. Therefore, an LVEF of 58% is a better cutoff value for reclassifying HFpEF patients based on cardiac SNS activation.

## Figures and Tables

**Table 1:** Clinical characteristics of study groups at discharge.

Characteristic	All Patients	LVEF ≥ 58%	LVEF < 58%	*p*-Value	LVEF ≥ 50%	LVEF < 50%	*p*-Value
Number (male %)	63 (49.2)	15 (46.7)	48 (50)	0.82	22 (45.5)	41 (51.2)	0.66
Age (years)	78.4 ± 9.6	82.5 ± 9.8	77.1 ± 9.3	0.06	79.8 ± 10.5	77.7 ± 9.2	0.40
Height (cm)	155.3 ± 9.1	155.9 ± 11.7	155.0 ± 8.2	0.75	155.2 ± 11.1	155.3 ± 7.9	0.96
Weight (kg)	52.0 ± 11.0	53.5 ± 11.3	51.6 ± 10.9	0.55	52.0 ± 11.4	52.0 ± 10.9	1.00
Body mass index (kg/m^2^)	21.5 ± 3.8	22.0 ± 4.1	21.3 ± 3.7	0.56	21.5 ± 3.8	21.5 ± 3.8	0.99
NYHA class	2.6 ± 0.7	2.4 ± 0.7	2.6 ± 0.7	0.35	2.5 ± 0.8	2.6 ± 0.7	0.66
Systolic BP (mm Hg)	114.0 ± 16.7	121.3 ± 13.1	111.7 ± 117.1	0.051	118.4 ± 14.0	111.6 ± 17.6	0.12
Diastolic BP (mm Hg)	65.8 ± 11.2	65.1 ± 11.5	66.0 ± 11.3	0.79	64.7 ± 10.7	66.3 ± 11.6	0.58
Heart rate (beats/min)	68.7 ± 11.0	63.9 ± 13.1	70.3 ± 10.0	0.050	66.1 ± 11.9	70.2 ± 10.4	0.17
LVEF (%)	44.6 ± 17.9	70.4 ± 8.3	36.6 ± 11.1	<0.001	65.4 ± 10.2	33.5 ± 8.9	<0.001
BNP (mg/dL)	310.9 [185.2–560.0]	204.2 [144.6–313.0]	326.2 [185.9–618.6]	0.10	196.4 [105.5–318.0]	342.6 [191.8–654.8]	0.01
HbA1c (%)	6.3 ± 0.8	6.0 ± 0.6	6.4 ± 0.8	0.20	6.2 ± 0.9	6.3 ± 0.8	0.48
Serum creatinine (mg/dL)	1.1 ± 0.8	1.1 ± 0.5	1.1 ± 0.8	0.76	1.0 ± 0.5	1.2 ± 0.9	0.34
eGFR (mL/min/1.73 m^2^)	54.3 ± 24.6	51.1 ± 17.2	55.3 ± 26.6	0.57	55.7 ± 20.0	53.5 ± 26.9	0.74
Hemoglobin (g/dL)	12.2 ± 2.0	11.7 ± 1.9	12.3 ± 2.1	0.32	11.8 ± 2.0	12.4 ± 2.1	0.22
Sodium (mEq/L)	140.0 ± 2.6	140.8 ± 1.5	139.7 ± 2.9	0.17	140.5 ± 2.2	139.7 ± 2.9	0.31

Data are expressed as mean ± standard deviation or number or frequency (%). BNP is represented by the median and interquartile range (IQR). NYHA, New York Heart Association; BP, blood pressure; LVEF, left ventricular ejection fraction; BNP, B-type natriuretic peptide; eGFR, estimated glomerular filtration rate. BNP, which is a non-normally distributed variable, was compared using the Mann-Whitney U test. Other normally distributed variables, were compared using an unpaired Student’s *t*-test.

**Table 2:** Comparisons of underlying disease.

Characteristic	All Patients	LVEF ≥ 58%	LVEF < 58%	*p*-Value	LVEF ≥ 50%	LVEF < 50%	*p*-Value
Etiology							
Ischemic CM (*n*, %)	12 (19.0)	0 (0)	12 (25)	0.03	2 (9.1)	10 (24.4)	0.14
Non-ischemic CM (*n*, %)	31 (49.2)	4 (26.7)	27 (56.3)	0.045	6 (27.3)	25 (61.0)	0.01
Hypertensive (*n*, %)	3 (4.8)	2 (13.3)	1 (2.1)	0.07	3 (13.6)	0 (0)	0.02
Valvular (*n*, %)	5 (7.9)	2 (13.3)	3 (6.3)	0.38	2 (9.1)	3 (7.3)	0.80
Abnormal heart rhythms (*n*, %)	12 (19.0)	7 (46.7)	5 (10.4)	0.002	9 (40.9)	3 (7.3)	0.001
Co-morbidity							
Hypertension (*n*, %)	27 (42.9)	8 (53.3)	19 (39.6)	0.35	13(59.1)	14 (34.1)	0.06
Diabetes mellitus (*n*, %)	17 (27.0)	3 (20)	14 (29.2)	0.49	5 (22.7)	12 (29.3)	0.58
Prior heart failure (*n*, %)	24 (38.1)	2 (13.3)	22 (45.8)	0.02	5 (22.7)	19 (46.3)	0.07
Atrial fibrillation (*n*, %)	38 (76.2)	13 (86.7)	25 (52.1)	0.02	18 (81.8)	20 (48.8)	0.01

Data are expressed as number or frequency. CM, cardiomyopathy. These data were compared using the chi-squared test.

**Table 3:** Comparisons of medication.

Characteristic	All Patients	LVEF ≥ 58%	LVEF < 58%	*p*-Value	LVEF ≥ 50%	LVEF < 50%	*p*-Value
Anti-platelet (%)	28.6	20.0	31.2	0.40	18.2	34.1	0.18
Anti-coagulants (%)	57.1	80	50	0.04	77.2	46.3	0.02
Diuretics (%)	69.8	53.3	75.0	0.11	59.1	75.6	0.17
Statins (%)	25.4	26.7	25.0	0.90	18.2	29.3	0.34
ACEIs (%)	39.7	13.3	47.9	0.02	22.7	48.8	0.04
ARBs (%)	22.2	20.0	22.9	0.81	22.7	22.0	0.94
AAs (%)	39.7	33.3	41.7	0.57	27.3	46.3	0.14
β-blockers (%)	66.6	53.3	70.8	0.21	50.0	75.6	0.04
Bisoprolol (%, mg)	30.2	26.7 (0.7 ± 1.4)	31.3 (0.6 ± 1.2)	0.83	31.8 (0.7 ± 1.3)	29.3 (0.6 ± 1.2)	0.82
Carvedilol (%, mg)	38.1	33.3 (1.5 ± 2.8)	39.6 (2.3 ± 4.0)	0.46	22.7 (1.2 ± 1.4)	46.3 (2.7 ± 4.2)	0.09
CCBs (%)	23.8	40.0	18.8	0.09	40.9	14.6	0.02

Data are expressed as frequency (%). Parentheses indicate mean ± standard deviation. ACEI, angiotensin-converting enzyme inhibitor; ARB, angiotensin receptor blocker; AA, aldosterone antagonist; CCB, calcium channel blocker. The frequency of medications used between the two groups was compared using the chi-squared test. Parentheses data were compared using an unpaired Student’s *t*-test.

**Table 4:** Differences in LVEF and cardiac SNS activity by gender.

	LVEF ≥ 58%			LVEF < 58%		
	Male (*n* = 7)	Female (*n* = 8)	*p*-Value	Male (*n* = 24)	Female (*n* = 24)	*p*-Value
LVEF (%)	69.9 ± 9.5	70.8 ± 7.7	0.85	34.9 ± 11.4	38.3 ± 10.8	0.29
Delayed H/M	2.1 ± 0.2	2.1 ± 0.5	0.90	1.8 ± 0.4	1.7 ± 0.3	0.41
WR (%)	29.3 ± 4.4	36.1 ± 16.6	0.32	41.6 ± 15.0	48.6 ± 15.3	0.11
	LVEF ≥ 50%			LVEF < 50%		
	Male (*n* = 10)	Female (*n* = 12)	*p*-value	Male (*n* = 21)	Female (*n* = 20)	*p*-value
LVEF (%)	65.6 ± 10.4	65.1 ± 10.5	0.91	31.9 ± 8.6	35.2 ± 9.0	0.24
Delayed H/M	2.0 ± 0.4	1.9 ± 0.5	0.57	1.8 ± 0.3	1.7 ± 0.4	0.64
WR (%)	30.8 ± 8.9	43.6 ± 18.1	0.06	42.6 ± 14.9	46.6 ± 15.6	0.40

Data are expressed as mean ± standard. LVEF, left ventricular ejection fraction; H/M, heart/mediastinum; WR, washout rate. These data were compared using an unpaired Student’s *t*-test.

**Table 5:** The number of patients who reached the study endpoint.

	Total Patients (*n* = 63)	LVEF ≥ 58% (*n* = 15)	LVEF < 58% (*n* = 48)	LVEF ≥ 50% (*n* = 22)	LVEF < 50% (*n* = 41)
Hospitalization due to heart failure	28	5	23	9	19
All-cause mortality	18	1	17	3	15
Cardiac death	10	0	10	1	9
Non-cardiac death	8	1	7	2	6

LVEF; left ventricular ejection fraction.
